# The impact of Septoria tritici Blotch disease on wheat: An EU perspective

**DOI:** 10.1016/j.fgb.2015.04.004

**Published:** 2015-06

**Authors:** Helen Fones, Sarah Gurr

**Affiliations:** Biosciences, College of Life and Environmental Sciences, University of Exeter, Stocker Road, Exeter EX4 4QD, UK

**Keywords:** Septoria tritici Blotch, Wheat, Crop yields, Economic value

## Abstract

*Zymoseptoria tritici* is the causal agent of one of the European Union’s most devastating foliar diseases of wheat: Septoria tritici Blotch (STB). It is also a notable pathogen of wheat grown in temperate climates throughout the world. In this commentary, we highlight the importance of STB on wheat in the EU. To better understand STB, it is necessary to consider the host crop, the fungal pathogen and their shared environment. Here, we consider the fungus *per se* and its interaction with its host and then focus on a more agricultural overview of the impact STB on wheat. We consider the climatic and weather factors which influence its spread and severity, allude to the agricultural practices which may mitigate or enhance its impact on crop yields, and evaluate the economic importance of wheat as a food and animal feed crop in the UK and EU. Finally, we estimate the cost of STB disease to EU agriculture.

## Highlights

• *Zymospetoria tritici* is a threat to wheat production in the EU Z.t.’s plastic genome increases the potential severity of this threat in the future.
• Climate change may also affect the risk from Z.t.
• We estimate the spore numbers produced by Z.t. during each infection cycle.
• We calculate 1) the economic value of wheat in the three main EU producers 2) the cost of and economic return for fungicide treatment of wheat vs Z.t.

## 1 Introduction

Septoria tritici Blotch (STB) poses a serious and persistent challenge to wheat grown in temperate climates throughout the world. This threat has triggered an intensive research effort to evaluate current disease control practices and to look for novel control strategies. Despite the huge economic importance of this pathogen (contained within this article and in Torriani et al., 2015), solid facts in peer-reviewed publications regarding yield losses or, indeed, the financial implications of disease are hard to find. For example, published losses due to STB disease recorded in one particular study in a defined geographic region have become widely adopted in the literature as being relevant to losses in all regions of the world (Eyal et al., 1973, 1987). Such extrapolations should not form the basis for economic, political and agricultural decision-making. In this article, we therefore set out to collate all available information, gathered from peer-reviewed scientific publications, publically accessible data-bases and web-sites, to paint a more realistic picture of the importance of STB in Europe. We hope that this merged information provides a solid basis with which we can evaluate the challenge of STB disease in Europe.

## 2 The importance of wheat as an EU crop

Wheat is the most widely-grown crop in the world. Global harvests reached 705 million metric tonnes (mmt) in 2013–2014 (www.agrimoney.com/). Within the EU, wheat advances from its world position of second most important food crop (after rice) to the status of most important cereal. In 2013/2014 the various countries which comprise the EU produced over 143 mmt of wheat; some 15% more than China, 35% more than India and 60% more than USA (calculations based on www.fao.org/worldfoodsituation/en/). Of the various EU member states, France and Germany are the biggest wheat producers, harvesting circa 26% and 17% respectively of the EU total, with UK gathering around 8.5% (Table 1). Over the past 10 years, EU metric tonnage has increased by 23%, whilst, over the same period, US tonnages have fallen by around 8% (faostat3.fao.org/).

The EU currently exports up to 15% of its harvest (ec.europa.eu/agriculture/cereals) and this figure is rising annually. Wheat grain grown in the EU provides calories for human foodstuffs (less than one third of harvest) and animal feed (circa two thirds of harvest). Wheat is also grown for alcohol distillation, as a raw material for biofuels and wheat straw is used for livestock bedding and fodder, roof thatching and basket-making.

Such figures and statistics attest to the huge economic and social importance of wheat as an EU crop and commodity. It follows that losses to the wheat crop from attack by pests and infection by pathogens are of considerable concern. Of the various pathogens, the foliar disease of wheat, Septoria tritici Blotch (STB), caused by the fungus *Zymoseptoria tritici*, is most problematic in our wheat fields (Shaw and Royle, 1989; Eyal et al., 1987). *Z*. *tritici* flourishes in the humid climate that prevails in EPPO’s “Maritime Zone” (Bouma, 2005 EPPO bulletin 35). This climatic region includes Northern France and Germany, as well as the UK. Thus, the fungus pervades the major wheat growing regions of the EU. In fact, this persistent pathogen accounts for approximately 70% of annual fungicide usage in the EU. During severe epidemics, losses of up to 50% of yield have been documented in fields planted with wheat cultivars susceptible to STB (Eyal et al., 1973: Eyal et al., 1987). In the UK, annual losses averaging around 20% of harvest are recorded when susceptible varieties on the 2012–2013 Home Grown Cereal Authority (HGCA) recommended list are deployed (www.hgca.com/media/.../ts113_septoria_tritici_in_winter_wheat.pdf) and are not treated with fungicides. However, smaller yield losses, of around 5–10%, are seen when wheat varieties are selected for disease resistance and when crops are sprayed with fungicide (hgca.com/.../g58-wheat-disease-management-guide-feb-2014). For the 25 wheat varieties recommended for autumn planting (winter wheat) HGCA give an average resistance score of 5 on a 1–9 scale in which high numbers indicate high resistance (HGCA recommended list^R^). Although this resistance is only partial, and thus some yield losses are still incurred, it has proven durable – advantageously – against all known fungal genotypes (Angus and Fenwick, 2008; Chartrain et al., 2015).

## 3 *Zymoseptoria tritici* as a threat to wheat production

The fungus, *Z*. *tritici*, has remained a relatively understudied pathogen (reviewed in: Kema et al., 1996; Duncan and Howard, 2000; Palmer and Skinner, 2002; Orton et al., 2011; Steinberg, 2015), particularly with regards to the paucity of molecular and cellular-based tools to interrogate the fungus *per se* (addressed in this issue). Moreover, our restricted knowledge of the interaction between the fungus and its host constrains our ability to optimise STB disease control strategies. For instance, this dimorphic pathogen exhibits an unusual and protracted ‘latent’ phase following its arrival on wheat (rev. in Orton et al., 2011). This phase describes a period when the fungus is associated with the leaf, but where the leaf exhibits no disease symptoms. Under field conditions in the summer this period persists for around 14 days, with this time protracted in colder weather up to 28 days (HGCA data). Whilst some very low level fungal activity has been described during this latent period (Keon et al., 2007; Shetty et al., 2007), a full understanding of its nutrient acquisition strategy and of its fine-scale temporal and spatial interactions with wheat remains elusive (Rudd et al., 2015; Sánchez-Vallet et al., 2015). Our state of knowledge regarding the molecular cross-talk between *Z*. *tritici* and the wheat immune system is elegantly summarised in O’Driscoll et al. (2014). Indeed, the timing of fungicide application is somewhat problematic, as it is difficult to match it confidently with disease progression. Fungicides sprayed at disease onset are effective for approximately seven of the 14–28 day latent period. Thus, whilst the leaves remain asymptomatic and the farmer considers that disease has been eradicated, it is possible that in fact the fungus proliferates. Best practise therefore requires that fungicide be sprayed early, before disease appears, to protect developing stem leaves, and again at ear emergence, to protect the flag and upper leaves (http://www.hgca.com/media176167/g63-wheat-disease-management-guide.pdf). However, if lesions begin to show on the leaves, STB has taken hold and fungicide application will be of limited utility. Few curative fungicides – chemistries that prevent pathogen colonisation of host tissues – are available. Those fungicides which are curative towards STB are among those to which resistance is developing (in particular, the azoles).

*Z*. *tritici* shows many characteristics typical of fungal plant pathogens – it has, for example, a mixed reproductive system and can generate large populations of spores (see below). Substantial gene flow can occur between fungal strains (Zhan and McDonald, 2004), with up to 30% of the *Z*. *tritici* population at the end of a growing season resulting from sexual reproduction (Eriksen et al., 2001) – a trait likely to decrease the time needed for it to adapt to control measures. Moreover, genome sequencing has revealed some unusual “hallmarks” in *Z*. *tritici* (Goodwin et al., 2011: Stukenbrock et al., 2010). The fungus carries 21 chromosomes, with thirteen core and eight dispensable chromosomes (meaning that they are supernumerary or accessory and can be lost without obvious effects on fungal fitness; commentary in Croll and McDonald, 2012). Around 17% of the genome is estimated to be repetitive. Of this, 70% is enriched in class 1 transposable elements (retro-elements which amplify *via* an RNA intermediate, thus introducing new mutations). The dispensable chromosomes carry a higher percentage of repetitive elements than the core chromosomes (Dhillon et al., 2014). Further, genes on these dispensable chromosomes which have homologues in the core chromosomes of sister species show an accelerated rate of evolution compared with these core genome homologues (Stukenbrock et al., 2011). The ability to dispense with up to eight chromosomes suggests, *a priori*, that this could hasten the loss of core fungicide target genes, that the fungus may develop resistance to fungicides (Torriani et al., 2009; Cools and Fraaije, 2013), alter its host-specificity (Stukenbrock et al., 2010) or, indeed, become able to overcome host disease resistance (Mundt et al., 1999, 2002; Rudd et al., 2015). Although these ideas are, at present, speculative, such suggestions merit investigation.

**Fig. 1:**
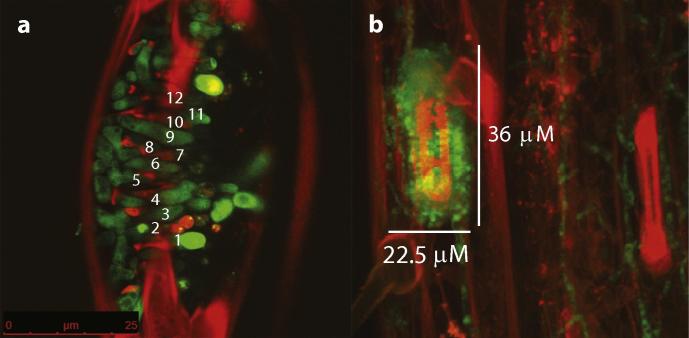
Spores per pycnidium. (a) Example image of maturing spores in a pycnidium (20 days post infection, *Z*. *tritici* IPO323 expressing cytosolic e-GFP (Kilaru et al., 2015) on wheat cv Galaxie; confocal microscopy with propidium iodide counterstaining); 12 spores can be counted in approximately half the length of the pycnidium, giving ∼25 spores along the length. (b) Example image (as a) showing a pycnidium at 21 dpi with measurements showing that length/width is approximately 1.6×. Approximately circular pycnidiospores are thus produced in an ellipse with length 25 and width 15.6 (spores), giving a cross sectional area of around 300 spores per pycnidium.

Modern agricultural practices have favoured the planting of vast hectarages of genetically uniform crops. Wheat fields in Europe are extensive and are planted with just a handful of cultivars moderately resistant to STB. Such practice favours the build-up of inoculum levels, so potentially hastening the emergence of new fungal pathotypes both from sexual reproduction between compatible strains (or indeed incompatible strains, see Kema et al., 2000) and via emergence of aggressive strains from a vast population of asexual spores. But how large are these fungal populations and what is the risk of new strains emerging? We can, for example, estimate the STB asexual spore load per hectare in a growing season. Planting densities for wheat are around 100 plants per m^2^, with each plant carrying 5 leaves. There are therefore up to 5 × 10^6^ wheat leaves receptive to inoculum per hectare. Assuming the asexual pycnidiospore generation to generation time is around 20 days, then, over a growing season, this polycyclic pathogen can cycle up to 6 times on a maturing crop. If the average disease rating for the wheat cultivars is 5 (HGCA listing) and infected wheat with this level of partial resistance generates 20 lesions per leaf (Jenna Watts, HGCA *pers*. *com*.) each 10 mm^2^, with 2 pycnidia per mm^2^ (see Fones et al., this issue), then a leaf could carry 400 pycnidia. Mature pycnidia fill the wheat substomatal cavity and carry approximately 300 spores (Fig. 1). Not all pycnidiospores would mature simultaneously nor be competent to infect but, from this, we can estimate that the asexual spore load per hectare over a growing season may reach 10^10–11^ spores. Whilst not all rain-splashed spores will cause infection, this high pathogen load, coupled with the “plasticity” of the *Z*. *tritici* genome, means that emergence of new fungal strains is of grave concern.

## 4 Environmental factors and their impact on the importance of a *Zymoseptoria tritici* as a wheat pathogen

Due to the economic importance of STB, various attempts have been made to understand and model the effects of weather on the incidence of this disease (Gladders et al., 2001; Pietravalle et al., 2003). As a result, the factors that alter the chance of an STB epidemic are quite well understood. Because *Z*. *tritici* requires a moist leaf surface for successful infection and is spread throughout the crop canopy *via* rain splash, the frequency of either very wet days (>10 mm rainfall) or consecutive wet days (three days with at least 1 mm rain) during the early growth of the wheat crop has been found to be of major importance in predicting outbreaks (hgca.com/publications/documents/7_2010_foliar_diseases.pdf). Similarly, the frequency of weather fluctuations is important, with temperatures below −2C in the early stages of growth reducing the risk of STB for winter wheat in the UK. te Beest et al. (2009a,b) have recently extended such weather-based models (Coakley et al., 1985; Shaw and Royle, 1993; Pietravalle et al., 2003) to take into account economic and environmental risk – the costs of planting resistant seeds and applying various fungicide doses over the growing period. This model informs choice amongst wheat growers to both guide and cut the costs of management of STB (te Beest et al., 2009b).

Finally, farming practices and altered weather patterns will influence STB disease severity. Firstly, the current trend for earlier autumn sowing of winter wheat in the UK, that is, in September and not in November or December (HGCA) is likely to increase the challenges posed by STB. Seedlings from early autumn planted seeds will endure temperatures more conducive to the establishment of infection and be exposed to sufficient rainfall to facilitate spread of pycnidiospores. In addition, this change will alter the period during which ascospores must survive on stubble between crops. This could be important – for instance, Daamen and Stol (1992) suggested that ascospore survival might play a large role in determining subsequent epidemic severity. Secondly, and conversely, climate change may reduce STB severity. Projections made for three different regions of France suggest a reduction of 2–6% in STB severity Gouache et al. (2013). Extrapolation of such findings across all wheat growing areas is, however, hampered by uncertainties regarding local effects of climate change on specific geographic areas.

Other disease management practices will be important in reducing the damage caused by STB: (i) Continuing breeding for resistance, particularly that which relies upon a broad genetic base, rather than single STB genes, will allow us to capitalise on recent advances in this area; (ii) improving STB forecasting abilities based upon more climate variables will better inform decision making with regards to the timing of fungicide application (te Beest et al., 2009b); (iii) better understanding of the “behaviour” of *Z*. *tritici* on and in the leaf, in terms of both leaf penetration and subsequent sporulation, will both inform fungicide choice and also timing of application; (iv) the practice of burning of above ground wheat stem residue/stubble post-harvest merits revisiting. Stubble-retention has been practiced in EU for the past 19 years for economic and smoke pollution reasons. However, stubble provides an ideal reservoir for *Z*. *tritici* survival (and that of other Dothideomycete plant-pathogens) between growing seasons, and for the build-up of inoculum to infect newly planted wheat. (v) Alternative host species need to be eradicated from the vicinity of the crop. *Z*. *tritici* has been recorded on 26 grasses, as collated by Suffert et al. (2011) and on one non-graminaceous weed (chickweed; Prestes and Hendrix, 1978). However, of these, only six grass species have been validated as alternative hosts of STB in at least two independent studies (Suffert et al., 2011). Thus, the risk posed by alternate hosts, and its importance, is currently unclear. A closer evaluation of the risk of this fungus “host hopping” from wheat onto volunteer grass species and then back onto new crops is merited.

## 5 Losses and future risks

Table 1 details the value of the wheat harvest to the individual economies of the three main EU wheat growing countries. Wheat is worth several billion Euros € to France (€ 7.2 billion Euros: $8.5 billion), Germany (€ 4.9 billion: $5.8 billion) and the UK (€ 2.4 billion: $2.8 billion).

Let us assume that disease mitigation – the planting of moderately disease resistant cultivars (see Brown *et al*., this edition) and application of a 3 spray fungicide regime (at a cost of 100 Euros per hectare, see Torriani et al., this edition) – is practiced uniformly across these three nations. Under these conditions, year-on-year wheat losses are between 5% and 10% (see above). The cost to each country’s economy is therefore twofold (Table 2); (i) the direct loss of the wheat harvest due to disease and (ii) the cost of fungicide application, with total cost reduced by the enhanced yields. 5–10% losses in France, Germany and UK give direct costs ranging between €120 and 700 million. Fungicide treatment additionally costs the farmers between €160 and 500 million across these three nations. Such costs are counter-balanced by boosted harvests post-spraying (estimated to be some 2.5 tonnes per hectare (see Torriani et al., 2015)). This returns between €800 and 2400 million into the economies of the three EU member states. Such estimates highlight two things; (i) the high return (some 2.5–7 fold) on investment by the farmer in terms of efficacy of chemical control (ii) the constant need for fungicide discovery, particularly in the face or emerging resistance to extant chemistries by a constantly evolving fungus.

## 6 In conclusion

• Wheat is of huge and growing societal and economic importance across the EU, both in terms of food and commodities.
• The most notable pathogen of wheat currently is the fungus *Z*. *tritici*, which causes Septoria tritici Blotch
• Our current understanding of *Z*. *tritici* is suggestive of a pathogen whose importance must not be underestimated. This fungus shows a degree of evolutionary “plasticity” which may allow it to keep pace with innovations in disease control with relative ease.
• A full understanding of the fungus *per se* and of its fine-scale interaction with wheat remains elusive. A precise and detailed catalogue of events leading to full infection would better inform models aimed at predicting disease outcome and better inform the timing of fungicide spraying.

## 7 Recommendations

• We need to raise awareness of growers, politicians and the general public as to the threat to local and global food security by pathogens such as *Z*. *tritici*.
• We need to garner more research funding and to train more plant pathologists to face such challenges.
• We need more interdisciplinary and international research, particularly in the fields of predictive and climate change modelling. Here, models are needed to (i) predict the movement of crops and pathogens in the face of climate change and (ii) inform the timing of crop spraying with antifungals. Intelligent use of fungicide mixtures and the timing of their application at low dose must be informed by lab.-based and field trials and by multi-parameter epidemiological modelling.
• We need to search for new sources of durable disease resistance to be introduced by introgression or GM into wheat and which do not reduce crop yields.
• We need to evaluate new low dose, environmentally benign, broad spectrum multi-target site antifungals. In addition, the regulatory framework for agrochemicals needs to assess the benefits of fungicides as well as their costs in order to achieve sustainable disease control.
• We need to reduce our reliance on single target site antifungals as resistance has emerged to these.

## Figures and Tables

**Table 1:** Wheat harvests and value in the 3 main EU wheat growing nations.

WHEAT – (2013 harvest)	France	Germany	UK
Hectares planted (million)	4.95	3.1	1.63
Yield: tonnes per hectare	7.4	8	7.4
Harvest total (m Tonnes)	37	24.9	12.1
Value per tonne (Euros)	195	195	195
Value to economy of named country (million Euros)	7200	4900	2400

Data from: Analyst Agritel; Agri.eu/wheat-market; Farming-statistics@defra; Federal ministry of food and agriculture; International grain council wheat index; Agrimoney.

**Table 2:** Value of losses due to STB and fungicide usage *per annum* in the major EU wheat growing nations.

Losses to STB^a^	France	Germany	UK
Value of 5–10% harvest losses due to STB in million Euros	350–700	250–500	120–240
Spraying costs, at 100 Euros per hectare^b^∗	459 × 10^6^	310 × 10^6^	163 × 10^6^
Added value in million Euros: assuming boost in crop yield of 2.5 tonnes per hectare, post spraying^c^∗	2400	1500	790

a Figures are given in Euros.

b The spraying costs equate with 3 fungicide sprays per season (see Torriani et al.⁎ 2015).

c Yield enhancements following fungicide sprays.
